# Saliva

**Published:** 1888-09

**Authors:** C. W. Berry

**Affiliations:** Ann Arbor, Mich.


### Saliva.

BY C. W. BERRY, D.D.S., ANN ARBOR, MICH.

Saliva is a commingled fluid or secretion which is a chemical product that results from the reaction of the blood upon the one hand, and the glands upon the other, being secreted or poured into the mouth by three sets of glands, namely, the parotid, the submaxillary, and the sublingual.

The parotid being the largest of these glands, lies between the ramus of the inferior maxilla and mastoid process of the temporal bone, and presents an extent of irregular surface from the zygomatic arch above to the angle of the lower jaw below, and from the mastoid process and meatus behind to the masseter muscle in front.

This gland is made up of many small lobules united together by cellular tissue covered externally by a dense fascia; its function is to secrete or separate from the blood the greater part of the saliva furnished to the mouth. The saliva is conveyed from this gland by the duct of steno or parotid duct, which has its opening into the mouth through the buccinator muscle and mucous membrane, opposite the upper second molar.

The secretion from this gland is a clear, watery, alkaline liquid, flowing in very small quantity when the mouth is at rest, and increasing greatly during the act of mastication or by artificial stimulation.

The submaxillary being next in size of these glands, is oval in form situated under and along the inferior edge of the body of the lower jaw, and is only separated from the parotid by a process of fascia. The secretion from this gland is conveyed through the duct of Wharton which enters the mouth below the tip of the tongue.

The sublingual the smaller in size of these glands is situated beneath the anterior and lateral portion of the tongue, and are covered by mucous membrane. The secretion from these glands is not conveyed by one duct, but through many smaller ones, which enter the mouth along the side of the tongue. Besides these three sets of glands mentioned as furnishing the oral secretion, the mucous membrane lining the whole interior of the mouth is furnished with small glands or openings, which are named according to their situation, and convey a secretion called mucous. The analysis of this fluid is governed by the components which enter into it, the age and habits and state of bodily health of the person from which it is taken, although a fair composition may be given as follows:

| | |
|---|---|
| Water - - - - | 995.14 |
| Organic Matter - - - - | 1.34 |
| Phosphate of soda, lime, and magnesia | .98 |
| Sulpho-cyanide of Potassium - - | 0.06 |
| Chloride of sodium and potassium - | .84 |
| Mixture of epithelium - - - | 1.64 |
| | 1000.00 |

The normal mixed saliva is a turbid bluish-white, and is devoid of odor or taste, having a specific gravity 1002 to 1009. Upon standing it forms a grayish-white deposit, which by examination with a microscope will be found to contain pavement epithelium, mucous corpuscles, and debris of food. Its reaction is alkaline and its alkalinity is increased during mastication and decreased during fasting, while after a prolonged fasting it may be found slightly acid. The quantity secreted daily by the healthy average man is from sixteen to eighteen ounces and this amount as it is secreted is made use of by assisting in the formation of the bolus of food, after having previously aided in its mastication. It also lubricates its passage into the stomach and aids the appreciation of taste, also by lubricating the surfaces of the mouth and teeth, it prevents the adhesion of viscid substances and permits the movement of rapid mastication.

Considering the saliva now in some of its abnormal conditions, it is sometimes rendered acid by some cause as dyspepsia, inflammation of the stomach, or by excessive acid in the system by any disease as rheumatism, consumption, etc., which produce both local and general effect upon other organs in the body. The lodgement of particles of food between the teeth and their retention and putrefaction there, leads to the formation of certain organic acids which not only destroy the alkalinity of the solution, but irritate the mucous membrane in the mouth which leads to disease and abnormal changes in its structure. In a mouth containing decomposing particles of food certain forms are likely to be present, and these or at least some of them, are to be regarded as the cause of disease. This condition of the oral secretion as to its acidity, can be determined by a simple test in the use of litmus paper, the paper being of a bluish color in itself, will sooner or later be changed to a red when brought in contact with the acid secretion. A more accurate test may be carried out for any special acid which may be suspected. The presence of sulphuric acid may be determined by adding to the saliva a solution of barium chloride which will at once produce a white precipitate.

The presence of nitric acid may also be accurately determined by the rising of red fumes upon boiling the saliva with copper filings. The presence of hydrochloric acid is plainly marked by the white precipitate which is formed when a specimen of such saliva be added to a solution of silver nitrate.

These being the more common forms of acid found in the abnormal saliva there have also been found traces of lactic acid, uric acid, and urea, urates, water, bile, and albumen, which can each be determined by special tests. In simple gastric disorders the solution is generally acid, which may be accounted for by increase change in the tissues, the epithelium of glandules being cast off so rapidly that a chemical change takes place in the cells, causing the secretion to appear acid. As to the formation and deposit of salivary calculus from these secretions it is proven beyond doubt, that when the fluid presents an acid condition, little if any deposit is formed, while if it be strongly alkaline the lime salts are held in solution, in the blood by carbonic acid, but on passing through the glands and ducts, and coming in contact with the air, the absence of pressure allows a portion of the carbonic acid to escape as carbonic acid gas, whereupon a precipitate is thrown down and lodged upon the rough surfaces about the teeth, particles of food, epithelium, and mucous becoming intermixed, the whole mass hardens, by some process, and so remains until removed by mechanical skill.

